# Iatrogenic ureteral injury in colorectal surgery: a retrospective cohort analysis of risk factors and preventive strategies

**DOI:** 10.1590/0102-672020260000028e1957

**Published:** 2026-08-31

**Authors:** Giovanna Savoy PAZIN, Helena da Cunha Lopes de LIMA, Marcelo Lopes de LIMA, Wagner Eduardo MATHEUS, Claudio Saddy Rodrigues COY, Raquel Franco LEAL, Maria de Lourdes Setsuko AYRIZONO

**Affiliations:** 1 Universidade Estadual de Campinas, Faculty of Medical Sciences, Department of Surgery, Coloproctology Unit – Campinas (SP), Brazil.; 2 Pontifícia Universidade Católica de Campinas – Campinas (SP), Brazil.; 3 Hospital Centro Médico de Campinas, Urology Service – Campinas (SP), Brazil.; 4 Universidade Estadual de Campinas, Faculty of Medical Sciences, Department of Surgery, Urology Service – Campinas (SP), Brazil.

**Keywords:** Colorectal neoplasms, Ureter, Iatrogenic disease, Colorectal surgery, Neoplasias colorretais, Ureter, Doença iatrogênica, Cirurgia colorretal

## Abstract

**Background:**

Iatrogenic ureteral injuries (IUI) are rare surgical complications in abdominopelvic surgeries, with an incidence varying between 0.15 and 1.0%.

**Aims:**

To evaluate the incidence of IUI in elective colorectal surgeries performed in a tertiary university hospital.

**Methods:**

This is a retrospective analysis of patients operated from 2004 to 2022, who presented IUI. Demographic data, underlying disease, predisposing factors, surgery access, location of the lesions and their characteristics, diagnosis time, treatment carried out, and follow-up were analyzed.

**Results:**

In the period, 2,312 abdominopelvic surgeries were performed, of which 1,998 were open and 314 were laparoscopic, with 19 IUI (0.82%). The mean age was 55.6 years, 57.9% were male, and 89.5% were white. The majority of patients were overweight (52.6%), and 73.7% had a history of abdominal surgery. Primary rectal adenocarcinoma was the most common disease (47.4%), followed by tumor recurrences (21.0%). IUI occurred in 1.91% of laparoscopic surgeries and 0.65% of open surgeries (p=0.053); patients with tumor recurrence presented more IUI than those with primary tumors or benign diseases (p=0.006). They were commonly observed in the left ureter (52.6%) and in the distal portion (89.5%), the main mechanism being the section (57.9%). Intraoperative diagnosis occurred in 12 patients (63.2%). IUI correction was predominant in ureteral reimplantation and end-to-end ureteral anastomosis. Postoperative complications were common (47.4%), and one patient died from causes unrelated to surgery.

**Conclusions:**

IUI presented low incidence in colorectal elective surgeries and were more frequent in surgeries for tumor recurrences, in the left ureter and the distal third. Early diagnosis with repair of the injury provided better results.

## ARTICLE HIGHLIGHTS

Iatrogenic ureteral injuries are rare complications in abdominal and pelvic surgeries but are associated with high postoperative morbidity.

They are most commonly observed in gynecological and colorectal procedures, mainly in cases of large tumors, previous surgeries, radiotherapy, infectious processes, and inflammatory bowel diseases.

Iatrogenic ureteral injuries must be recognized intraoperatively and treated promptly. Late recognition worsens the prognosis and can lead to complications such as fistulas, abscesses, and loss of kidney function.

## Summary

**CENTRAL MESSAGE:**

Iatrogenic ureteral injuries (IUI) are important surgical complications with high morbidity but are rare in abdominal and pelvic surgical procedures, with an incidence varying between 0.15 and 1.0%. Their incidence is higher in gynecological surgeries, followed by colorectal operations. The incidence increases in individuals with previous pelvic surgeries, radiotherapy, inflammatory and/or infectious processes, inflammatory bowel disease, and urogenital abnormalities. Other factors that can increase the risk of ureteral injury are bleeding in the operative procedure, making it difficult to identify the ureter, especially in areas with poor visibility and with the presence of advanced tumors. IUI occurs more frequently during proctectomies (abdominoperineal resections and low anterior resections) and sigmoidectomies.

**PERSPECTIVES:**

Iatrogenic ureteral injuries had a low incidence, being more prevalent in males and overweight individuals. Rectal cancer, tumor recurrence, and neoadjuvant treatment were risk factors for the lesions. They were most commonly observed in the distal portion of the left ureter, with the section being the most prevalent mechanism. The majority was diagnosed intraoperatively, and the most commonly performed corrections were ureteral reimplantation and primary anastomosis, with patients presenting relatively high morbidity. An early diagnosis, preferably intraoperatively, is essential to improve treatment results, avoiding serious complications that can lead to loss of renal function requiring dialysis and/or nephrectomy.

## VISUAL ABSTRACT

**Figure:**
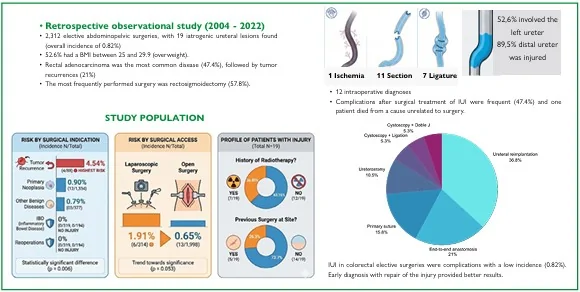

## RESUMO

**Racional:**

Lesões ureterais iatrogênicas (LUI) são complicações cirúrgicas raras em operações abdominopélvicas, com incidência variando entre 0,15 e 1,0%.

**Objetivos:**

Avaliar a incidência de LUI em cirurgias colorretais eletivas realizadas em um hospital universitário terciário.

**Métodos:**

Trata-se de uma análise retrospectiva de pacientes operados entre 2004 e 2022, que apresentaram LUI. Foram analisados os dados gerais dos pacientes, doença de base, fatores predisponentes, acesso cirúrgico, localização das lesões e suas características, tempo de diagnóstico, tratamento realizado e seguimento.

**Resultados:**

No período, foram realizadas 2.312 cirurgias abdominopélvicas, sendo 1.998 abertas e 314 laparoscópicas, com 19 LUI (0,82%). A média de idade foi de 55,6 anos, 57,9% eram do sexo masculino e 89,5% eram brancos. Adenocarcinoma primário do reto foi a doença mais comum (47,4%), seguido por recidivas tumorais (21,0%). Ocorreram LUI em 1,91% das cirurgias laparoscópicas e 0,65% das cirurgias abertas (p=0.053); pacientes com recidiva tumoral tiveram mais LUI do que aqueles com tumor primário ou doenças benignas (p=0.006). LUI foram mais comumente observadas no ureter esquerdo (52,6%) e na porção distal do órgão (89,5%), sendo o principal mecanismo a secção (57,8%). Diagnóstico intraoperatório ocorreu em 12 pacientes (63,2%). Na correção das LUI, houve predomínio de reimplante ureteral e anastomose ureteral término-terminal. Complicações pós-operatórias foram comuns (47,4%), e um paciente faleceu por causas não relacionadas à cirurgia.

**Conclusões:**

As LUI apresentaram baixa incidência em cirurgias colorretais eletivas e foram mais frequentes nas operações para recidiva tumoral, no ureter esquerdo e no terço distal. O diagnóstico precoce com reparo da lesão proporcionou melhores resultados.

## INTRODUCTION

 Iatrogenic ureteral injuries (IUI) are important surgical complications with high morbidity but are rare in abdominal and pelvic surgical procedures, with an incidence varying between 0.15 and 1.0%^31^. Their incidence is higher in gynecological surgeries, followed by colorectal operations^2,14,25,42^. This can partly be explained by the proximity of the ureter to the areas of dissection of the female reproductive system and the rectosigmoid. The distal ureter is the most vulnerable region for iatrogenic injuries, corresponding to 61 to 90% of cases, and 74% of them occur on the left side^27,37^. 

 Although most IUI occurs in individuals without significant risk factors^11^, its incidence increases in individuals with previous pelvic surgeries, radiotherapy, inflammatory and/or infectious processes, inflammatory bowel disease (IBD), and urogenital abnormalities^17,18,24^. Other factors that can increase the risk of ureteral injury are bleeding in the operative procedure, making it difficult to identify the ureter, especially in areas with poor visibility, the presence of advanced tumors, as well as surgeons in the learning process, as in university hospitals^5^. 

 In colorectal cancer (CRC), the approach to large tumors in the pelvis, the involvement of lymph nodes or tumor implants, the requirement for extensive dissection to obtain adequate oncological margins, and patient malnutrition can lead to IUI^19,44^. IUI occurs more frequently during proctectomies (abdominoperineal resections and low anterior resections) and sigmoidectomies^44^, with an incidence of 0.3 to 5.7% of injuries in these types of procedures^16,41^. 

 This study aimed to evaluate IUI in elective surgeries in the Colorectal Unit of a tertiary university hospital. 

## METHODS

 Retrospective observational study of patients operated on electively by the Colorectal Unit in the University Hospital at the Universidade de Campinas (Unicamp) who presented with IUI from February 2004 to February 2022. Patients operated on for CRC, IBD (ulcerative colitis and Crohn’s disease), familial adenomatous polyposis, desmoid tumors, diverticular disease of the colon, and other benign diseases were included. 

 The following variables were analyzed: demographic data (sex, age, color, and body mass index [BMI]), underlying disease, predisposing factors (radiotherapy, previous surgeries), surgical access (open or laparoscopic), location of injuries and their characteristics (ligatures, sections), time of diagnosis (intraoperative or late), surgical treatment, complications of the urological procedure, and follow-up. Quantitative data were compared using Student’s T-test and categorical data using χ^2^ or Fisher’s exact test. 

 The treatment of IUI was carried out by the Urology team at the Service, and early follow-up was performed jointly by the Urology and Colorectal Unit teams. The present study was approved by the Unicamp Research Ethics Committee (CAAE: 66113222.8.0000.5404). 

## RESULTS

 During the study period, 2,312 elective abdominopelvic surgeries were performed by the Colorectal Unit. Nineteen IUI were recorded, corresponding to 0.82% of surgeries. The mean age of the patients was 55.6 (33–73) years, 11 (57.9%) were male, and the majority (89.5%) were white. More than half of the patients (52.6%) had a BMI between 25 and 29.9 (overweight), and the vast majority (73.7%) had previous abdominal surgery (Table 1). 

**Table 1:** General and demographic data.

Data		Patients n (%)
Sex		
	Male	11 (57.9)
	Female	8 (42.1)
Age		
	30–40	3 (15.8)
	41–50	5 (26.4)
	51–60	3 (15.8)
	61–70	4 (21.0)
	71–80	4 (21.0)
Color		
	White	17 (89.5)
	Black	2 (10.5)
Body mass index		
	<18,5	1 (5.3)
	18,5–24,9	6 (31.6)
	25–29,9	10 (52.6)
	30–34.9	2 (10.5)
	35–39,9	0
Previous abdominal surgeries		
	None	5 (26.3)
	Derivative colostomy	3 (15.8)
	Hartmann surgery	2 (10.5)
	Partial colectomy	2 (10.5)
	Total colectomy	1 (5.3)
	Abdominoperineal resection of the rectum	1 (5.3)
	Gynecological	2 (10.5)
	Others	3 (15.8)

 Rectal adenocarcinoma was the most common disease among patients with IUI (47.4%), followed by tumor recurrences (21.0%). Other indications for surgery included sigmoid neoplasms, post-Hartmann reconstruction of intestinal transit, and a presacral cystic lesion. Seven patients (36.8%) received neoadjuvant chemotherapy, and in six, radiotherapy was associated with chemotherapy. 

 The most commonly performed surgeries were rectosigmoidectomy (11; 57.9%), post-Hartmann reconstruction (2; 10.5%), and abdominoperineal resection of the rectum (2; 10.5%). Regarding the incidence of IUI due to the underlying disease, the highest incidence was observed in surgeries for tumor recurrence, corresponding to 4.54%. In surgeries for primary neoplasia, the IUI rate was 0.90%, and in benign diseases, 0.79%. No IUI was observed in surgeries for IBD and reoperations (Table 2). A statistically significant association was observed between the number of lesions and the underlying disease (p=0.006; χ^2^ test), with patients tumor recurrences presenting more lesions than neoplasms and benign lesions. 

**Table 2:** Ureteral injuries and clinical and surgical characteristics.

N/Total (%)			p-value
Indication of the surgery			0.006
	Primary neoplasia	12/1.334 (0.90%)	
	Inflammatory bowel disease	0/319	
	Other benign diseases	3/377 (0.79%)	
	Reoperations	0/194	
	Tumor recurrences	4/88 (4.54%)	
Radiotherapy			----
	Yes	7/19 (36.85%)	
	No	12/19 (63.15%)	
Access			0.053
	Open surgery	13/1.998 (0.65%)	
	Laparoscopic surgery	6/314 (1.91%)	
Previous surgery			----
	Yes	14/19 (73.7%)	
	No	5/19 (26.3%)	

 Of the total of 2,312 surgeries, 1,998 (86.4%) were performed by laparotomy and 314 (13.6%) were laparoscopic procedures. Regarding IUI, six injuries (1.91%) were identified in laparoscopic surgeries and 13 (0.65%) in open surgeries. No statistically significant association was observed between the number of lesions and the surgical access route (p=0.053; χ^2^ test). 

 In patients with previous abdominal surgery, the occurrence of IUI was 73.7%, compared to 26.3% in those who did not have previous abdominal surgery (Table 2). More than half of IUI (52.6%) involved the left ureter; 36.8% were in the right ureter, and in two cases, bilateral injuries occurred (10.5%). In 89.5% of patients, the distal ureter was injured. The section was the most common mechanism of injury, corresponding to 11 cases (57.9%), followed by seven ligatures, and one ischemia (Table 3). 

**Table 3:** Characteristics of ureteral injuries.

		Patients N (%)
Laterality		
	Right	7 (36.8)
	Left	10 (52.6)
	Bilateral	2 (10.5)
Location		
	Distal ureter	17 (89.5)
	Proximal ureter	2 (10.5)
Type of injury		
	Ligature	7 (36.9)
	Complete grade iv	4 (21.1)
	Section >50% (grade iii)	3 (15.8)
	Section <50% (grau ii)	2 (10.5)
	Complete grade v	2 (10.5)
	Ischemia	1 (5.3)

 The diagnosis was made during surgery in 12 patients (63.2%), whereas in the remaining, it was postoperative and generally characterized by urinary fistulas. Of the late diagnoses, four patients had the lesion identified in less than a week, but in two of them, the diagnosis of IUI was only made two months after surgery. Of the injuries identified intraoperatively, four were treated with primary end-to-end ureteroureteroanastomosis, three with ureteral reimplantations (two using the psoic bladder technique and one using the Politano technique), three with primary sutures, one with ligation with a contralateral double J catheter, and one with ipsilateral nephrectomy (Table 4). 

**Table 4:** Surgical treatment performed for ureteral injuries.

Type of surgery	Patients N (%)
Ureteral reimplantation	7 (36.8)
End-to-end anastomosis	4 (21.0)
Primary suture	3 (15.8)
Ureterostomy	2 (10.5)
Cystoscopy + Ligation	1 (5.3)
Cystoscopy + Double J	1 (5.3)
Nephrectomy	1 (5.3)

 Of the seven late-diagnosed injuries, four patients underwent ureteral reimplantation (two using the Lich-Gregoir technique, one using the Boari flap, and one using the Politano technique); in two, ureterostomies were performed due to the impossibility of bladder reimplantation, and in one of them it was possible to insert a double J catheter without the need for an open surgical approach (Table 4). 

 Almost half of the patients (47.4%) had complications. Regarding surgical complications, three patients developed urinary fistulas, two of whom underwent reoperation, with a ureterostomy performed in one and Bricker surgery in the other; in the third, there was spontaneous closure of the fistula. Another patient underwent reoperation due to an enterocutaneous fistula and presented a new ureteral injury, which was treated with primary suture. However, he developed a highoutput urinary fistula, requiring a subsequent nephrectomy. There was one case of death due to complications not directly related to the surgical procedure. 

 In late follow-up, seven patients remain under follow-up, one of who is undergoing chemotherapy due to the progression of liver metastases. The others are asymptomatic and have no evidence of neoplasia or late complications resulting from IUI. Seven additional patients died due to the progression of the underlying disease. Five were lost to follow-up, and in three of them, progression of the neoplasm was noted in the medical record. 

## DISCUSSION

 IUI are surgical complications mainly observed in gynecological, colorectal, and vascular surgeries, in addition to urological endoscopic procedures. They are rare but present considerable morbidity, especially when the diagnosis is late^25^. In this study, the overall rate of IUI was 0.82%, similar to that reported in the literature^19,27^. 

 In the last 20 years, an increase in the incidence of IUI has been observed^26,32^. Although it is difficult to determine the exact reason, it appears to be associated with more complex cases operated^23,32^. Halabi et al.^19^, evaluating the occurrence of IUI over the 2001–2010 decade using a database from the United States, found an IUI rate of 0.28% (6,027 ureteral injuries in 2,165,848 colorectal procedures), which was more frequent in the second half of the decade. IUI were independently associated with higher mortality, morbidity, longer length of stay, and higher hospital costs. Risk factors included: rectal cancer, adhesions, metastatic cancer, malnutrition, and treatment at a teaching hospital; protective factors included the use of laparoscopy, transverse colectomy, and right colectomy^34^. 

 In the treatment of extraperitoneal rectal cancer, there is currently a consensus on neoadjuvant treatment for locally advanced tumors^38^. The surgical procedure in an irradiated pelvis can become more difficult due to adhesions and tissue fragility, increasing the chances of ureteral injuries during their dissection^19,40^. In the multivariate analysis carried out by Andersen et al.^3^, it was demonstrated that individuals undergoing neoadjuvant treatment, when adjusted for other demographic factors, have a 95% chance of having IUI, corroborating the data from the present study, in which 36.8% of patients with neoplasia who underwent neoadjuvant treatment had IUI, compared to 26.3% of patients with neoplasia who did not undergo chemotherapy and radiotherapy preoperatively. 

 As in other studies^17-19,37^, the highest rates of IUI were related to rectal cancer. A higher rate of IUI was observed in surgeries for tumor recurrence, where this complication occurred in 4.54% of patients, while in surgeries for primary neoplasia, this rate was only 0.9%. No IUI occurred in IBD surgeries in this sample, unlike in the study by Palaniappa et al.^31^, in which 14 IUI were identified during a 5-year prospective evaluation at a center in New York, half of which were in IBD surgeries. The prevalence of IUI in patients undergoing previous abdominal surgery was 73,7%, in contrast to only 26.3% of patients without any previous surgical approach, thus demonstrating that the risk of IUI also increases in patients with previous abdominal surgeries due to a *greater* number of adhesions. 

 There is no consensus regarding the assessment of demographic data in literature studies. In the present study, the predominance of lesions was in males (57.9%), unlike most studies, in which there is a predominance of IUI in females. The vast majority of these studies considered ureteral injuries that occurred during gynecological procedures, which makes female patients more prevalent. Palaniappa et al.^31^ and Halabi et al.^19^ attribute a possible explanation for this to female abdominal and pelvic anatomy. When evaluating only colorectal surgeries, as in the study by Andersen et al.^3^, the predominance was male, corroborating the findings herein. 

 The analysis also demonstrated similarity with other studies concerning age and BMI at the time of IUI. It is known that in obese patients, the anatomy and course of the ureter can be altered because of retroperitoneal fat that moves the ureter to a more medial position^18^. Kominsky et al.^22^, in their 10-year retrospective analysis of 87 patients who presented IUI in non-urological surgeries, reported a BMI was 29.9 kg/m^2^ in patients with an intraoperative diagnosis and 31.6 kg/m^2^ in those with a postoperative diagnosis, similar to the present study ,in which the majority of patients were overweight. 

 Although the advantages of laparoscopy over open surgery are well established, considering earlier recovery and shorter hospital stay, no study has adequately assessed whether the risk of ureteral injury differs significantly between the two approaches^27,31^. Some studies observed an increase in the incidence of IUI in laparoscopic colorectal surgery^23,27^, while others did not show this association^4,16,36^. 

 Regarding videolaparoscopic surgery, a higher incidence of IUI (1.91 *vs.* 0.65%) was observed, but there is no agreement among the studies found in the literature. Mayo et al.^28^ demonstrated in their retrospective analysis of colorectal surgeries performed over ten years and recorded in the Nationwide In-Patient Sample (NIS), the largest database of hospitalized patients in the United States, that minimally invasive surgery had a protective effect compared with open surgery. Besides, the incidence of injuries did not change over time, opposing the hypothesis that the increase in IUI in laparoscopies occurs because of the lower usage of invasive surgical techniques. Another factor that possibly explains the higher incidence of urinary tract injuries in minimally invasive procedures is the learning curve phenomenon. Chalya et al.^8^ demonstrated a higher incidence of IUI in patients undergoing abdominal and pelvic surgeries in peripheral hospitals where the procedures were performed by general surgeons with limited experience in performing more complex surgeries. 

 Andersen et al.^3^ evaluated 18,474 patients undergoing curative surgical resection of CRC (12,183 open and 6,291 laparoscopic surgeries) and observed 82 ureteral injuries (0.44%), with rates of 0.59% in the laparoscopy group and 0.37% in the open. Regarding colon cancer, no difference in the incidence of IUI was observed between the groups. In rectal cancer, the occurrence of ureteral injury was 1.00% in the laparoscopic approach and 0.42% in open surgery. In the multivariate analysis, adjusted for age, sex, ASA classification (American Society of Anesthesiologists), BMI, tumor staging, preoperative radiotherapy and chemotherapy, year of surgery, and surgeon’s specialty, only the laparoscopic approach was associated with an increase in the risk of ureteral injury. Conversely, Zafar et al.^46^ observed more ureteral injuries in open surgeries than in laparoscopies (0.66 vs. 0.53%). They analyzed 94,526 colectomies for benign, malignant, and inflammatory diseases, with the occurrence of IUI in 1.77% (585 patients among the 33,092 laparoscopy). Patients with ureteral injury had more septic complications and longer hospitalization. In a metaanalysis of 46 studies involving 491,519 patients undergoing gynecological or colorectal surgeries, Yanagisawa et al.^43^ found 1,719 ureteral injuries. Laparoscopic hysterectomy for cervical and endometrial cancer was associated with a higher rate of IUI than open surgery, but the same was not observed for laparoscopic colectomy for other diseases such as diverticulitis, CRC, and IBD. 

 Ureteral injuries can result mainly from inadvertent ligation or laceration, which can be partial or complete^33^. However, crushing or compression with surgical instruments, thermal injury due to coagulation, electric current, denervation, and devascularization can also be causes of IUI, which do not lead to immediate changes but can result in postoperative fistulas and stenosis^11,40^. Chalya et al.^8^ identified ligation in 36.6% of cases as the most common mechanism of IUI, unlike Bašić et al.^5^, who reported a higher incidence of partial transections (41.8%), followed by partial perforations (23.6%), complete transections (20.0%), and ligations (12.7%). Similar results to those found by Bašić et al.^5^ were observed, with a total of 57.9% of cases of section, contrasting with 36.8% of cases of ligation. The majority of the lesions were in the distal portion of the left ureter, as in other studies in the literature^27,39^. However, Chalya et al.^8^, in a retrospective study carried out at Bugano Medical Center, related a bilateral lesion rate of 20.4% of bilateral lesions, a higher percentage than observed in the present study, which was 10.5%. 

 IUI can be detected intraoperatively or postoperatively, but the most determining factor in the outcome of treatment is the interval between injury and repair; the longer the interval, the worse the outcome, with increased complications and loss of kidney function^31^. Unfortunately, around 50 to 70% of IUI are diagnosed late^27^. Recognition and repair of the injury acutely and immediately, or at most within a week after the injury, allows better results and fewer complications^2^. In our series, 63.2% of cases were diagnosed intraoperatively and, among those diagnosed late, the diagnosis was made after 60 days in only two cases. These patients developed urinary fistulas and impaired renal function, requiring ureterostomy in one patient and multiple surgical procedures in the other, both associated with prolonged hospital stays. 

 The use of a prophylactic ureteral catheter in colorectal surgery, especially in the videolaparoscopic approach where palpation of the ureters is not possible, is of great help in identifying them, as demonstrated in a systematic review of 26 studies published between 2000 and 2022 by Brollo et al.^7^. However, this procedure is associated with increased surgical time, more expenses, and adverse events such as ureteral obstruction and urinary tract infection^7,20,39^. Their indications are not clearly established in the literature, but they are generally recommended in cases of reoperation, large tumors, previous radiotherapy, diverticulitis, fistulas, Crohn’s disease, and obesity^7,13,20,30^. Other authors also recommend the use of a lighted ureteral catheterd or the use of fluorescence devices with intravenous injection of methylene blue or indocyanine green to better identify the ureters intraoperatively^6,35,45^. 

 Additionally, in a systematic review including 22 studies, Croghan et al.^10^ evaluated 869,603 patients (102,370 with stent and 767,233 without), where IUI occurred in 1.49% of the catheter group and 0.17% of the non-catheter group; intraoperative recognition of the injury was 62.50% in the first and 52.94% in the second group. The use of the catheter had a low rate of complications, but data were insufficient to conclude whether its prophylactic use reduces ureteral injury or increases the chance of identifying it, as in the systematic review and meta-analysis by Hird et al.^21^. Mazzarella et al.^29^, also in a systematic review involving 12 studies, analyzed the effectiveness of the technique using lighted catheters and fluorescent dyes for real-time intraoperative visualization of the ureter in minimally invasive surgeries. They observed an IUI incidence rate of 0.33% (out of a total of 822 patients who had ureteral identification), but no cases of injury occurred with fluorescent dye-guided surgery, although urinary tract infection (2.0%) and kidney injury (1.0%) were reported. Yanagisawa et al.^43^ compared the prophylactic ureteric stenting in gynaecological and colorectal surgeries. Although it was associated with a lower incidence of ureteric injuries during gynaecological surgery, it did not reduce the risk of ureteric injuries during colorectal surgery, directing its use to specific types of procedure. 

 Early diagnosis and repair of the injury during primary surgery can result in reduced morbidity and facilitate repair, improving outcomes. However, less than a third of injuries are diagnosed in the primary procedure^9,39,44^. An undiagnosed or inadequately repaired injury can lead to serious complications such as urinoma, abscess, ureteral stricture, ureteral fistula, and potential loss of the ipsilateral kidney^1^. Understanding the characteristics of each type of injury, as well as the position and blood supply of the ureter, is important because the etiology, in part, dictates the treatment approach^16^. When approaching the techniques used to treat IUI, one must take into account principles of mobilization and debridement, tension-free anastomosis, and passage of a double J catheter^27^. The passage of the double J ureteral stent is the first-line treatment for simple injuries, and it is an endoscopic procedure with a high success rate (87.5%), as documented in the multicenter retrospective analysis of clinical data from 22 patients with IUI in the Hospital of Caen, France, by Souli et al.^39^. The present study shows a diversity of techniques, but in all cases, treatment was performed by the Urology team and management was based on the location and extent of the lesion. 

 Injuries in short distal segments can be treated by reimplantation or ureteroureterostomy, while in longer strictures, reimplantation in the psoic bladder and Boari flaps can leave tension free. Interpositions of a segment of the gastrointestinal tract and nephrectomy are exceptional procedures^15^. In the patients evaluated, it was possible to identify a similar approach since the majority of complete and distal lesions were treated with ureteral reimplantation in the psoic bladder, while partial distal lesions were treated with end-to-end sutures/anastomoses. In cases of late diagnosis, minimally invasive ureteroscopic treatment can be attempted with the aim of dilation and passage of a double J catheter^12^, as was undertaken in one of the patients, in whom cystoscopy was performed with passage of this catheter, with resolution of the fistula. 

## CONCLUSIONS

 IUI had a low incidence in the sample, being more prevalent in males and overweight individuals. Rectal cancer, tumor recurrence, and neoadjuvant treatment were risk factors for the lesions. They were most commonly observed in the distal portion of the left ureter, with the section being the most prevalent mechanism. The majority was diagnosed intraoperatively, and the most commonly performed corrections were ureteral reimplantation and primary anastomosis, with patients presenting relatively high morbidity. The limitations of this study are it is retrospective design and the relatively small number of patients with IUI included, because it is considered a uncommon affection. An early diagnosis, preferably intraoperatively, is essential to improve treatment results, avoiding serious complications that can lead to loss of renal function requiring dialysis and/or nephrectomy. 

## Data Availability

The datasets generated and/or analyzed during the current study are available from the corresponding author upon reasonable request. The information regarding the investigation, methodology and data analysis of the article is archived under the responsibility of the authors.
